# Characterization of Enterococci- and ESBL-Producing *Escherichia coli* Isolated from Milk of Bovides with Mastitis in Egypt

**DOI:** 10.3390/pathogens10020097

**Published:** 2021-01-21

**Authors:** Wedad Ahmed, Heinrich Neubauer, Herbert Tomaso, Fatma Ibrahim El Hofy, Stefan Monecke, Ashraf Awad Abd El-Tawab, Helmut Hotzel

**Affiliations:** 1 Friedrich-Loeffler-Institut, Institute of Bacterial Infections and Zoonoses, Naumburger Str. 96a, 07743 Jena, Germany; heinrich.neubauer@fli.de (H.N.); herbert.tomaso@fli.de (H.T.); helmut.hotzel@fli.de (H.H.); 2 Department of Microbiology, Faculty of Veterinary Medicine, Benha University, Moshtohor Toukh P.O. Box 13736, Benha 13511, Egypt; fatmaelhofy@yahoo.com (F.I.E.H.); ashraf.awad@fvtm.bu.edu.eg (A.A.A.E.-T.); 3 Leibniz Institute of Photonic Technology (IPHT), Albert-Einstein-Str. 9, 07745 Jena, Germany; stefan.monecke@leibniz-ipht.de; 4 InfectoGnostics Research Campus Jena e. V., Philosophenweg 7, 07743 Jena, Germany

**Keywords:** enterococci, *Escherichia coli*, resistance gene, DNA microarray, mastitis, Egypt

## Abstract

This study aimed to investigate the prevalence and antimicrobial resistance of enterococci- and ESBL-producing *E. coli* isolated from milk of bovine mastitis cases in Egypt. Fifty milk samples of dairy animals were collected from localities in the Nile Delta region of Egypt. Isolates were identified using MALDI-TOF MS, and antibiotic susceptibility testing was performed by the broth microdilution method. PCR amplifications were carried out, targeting resistance-associated genes. Seventeen *Enterococcus* isolates and eight coliform isolates could be cultivated. Vancomycin resistance rate was high in *Ent. faecalis.* The VITEK 2 system confirmed all *E. coli* isolates as ESBL-producing. All *Ent*. *faecalis* isolates harbored *erm*(B), *tet*L and *aac-aph*D genes. The *van*A gene was detected in *Ent. faecalis* isolate, *van*B was found in other *Enterococcus,* while one isolate of *E. casseliflavus* exhibited the *van*A gene. *E. coli* isolates exhibited high prevalence of erm(B) and tetL. *E. coli* isolates were analyzed by DNA microarray analysis. Four isolates were determined by O-serotyping as O8 (n = 1), O86 (n = 2) and O157 (n = 1). H-serotyping resulted in H11, H12, H21 (two isolates each) and one was of H16 type. Different virulence-associated genes were detected in *E. coli* isolates including *lpf*A, *ast*A, *cel*B, *cma* *hem*L, *int*I1 and *int*I2, and the *iro*N gene was identified by DNA microarray analysis.

## 1. Introduction

Bovine mastitis is the most common and costly disease affecting dairy cattle throughout the world [1]. It leads to a reduction of the amount and quality of milk produced, increasing of veterinary costs and culling of severely infected animals [2].

Coliform bacteria are the most common pathogens that have been isolated from bovine mastitis [3]. The most important coliform pathogen is *Escherichia* (*E*.) *coli* that causes mastitis with high incidence in comparison to other coliforms. Especially, strain type O157:H7 is of great importance because of its zoonotic character [4].

Beside mastitis, *E. coli* is the most common pathogen responsible for various severe gastrointestinal or urinary tract infections, and even bacteremia in humans, causing thousands of deaths worldwide every year. Emergence of extended-spectrum β-lactamase (ESBL)-producing *Enterobacteriaceae*, especially *E. coli*, has been increased over the last decades. Possibly, a cause is the usage of cephalosporins as preferred agents to avoid mastitis-associated economic losses in dairy cattle [5]. High prevalence of ESBL-producing *E. coli* in animals has been demonstrated in many studies and varies between countries and animal species [6].

Enterococci are one of the environmental causative agents of mastitis. These opportunistic bacteria are part of normal physiological gut flora in humans and animals, but over the last years, they have become one of the main pathogens causing numerous infections in humans, mainly those hospital-acquired, such as bacteremia and infections of the urinary tract, skin, soft tissue, abdomen and pelvis and central nervous system. These infections are caused mainly by *Enterococcus (Ent.) faecalis* (about 80.0%) and *Ent. faecium* (10.0–15.0%). The high tolerance of enterococci to disadvantageous conditions allows their long survival in the environment, including in abattoirs [7]. In addition to their importance in causing diseases, enterococci can evolve resistance to antibiotics, and additionally to their intrinsic resistance properties. Enterococci may harbor multidrug resistance determinants for antimicrobial agents such as cephalosporins and aminoglycosides [8]. Generally, bacteria can produce antibiotic resistance with remarkably new mechanisms [9]. Resistance genes are often easily transferred to other species through conjugative transposons and plasmids, showing a broad host profile [10].

Due to the little information known about enterococci and *E. coli* from mastitis in bovides in Egypt, the aim of this study was to investigate the prevalence and antimicrobial resistance of enterococci and ESBL-producing *E. coli* isolated from milk of bovine mastitis cases in Egypt. The study completes the first part regarding staphylococci and streptococci from milk [11].

## 2. Materials and Methods

### 2.1. Sample Collection and Cultivation

The present study was carried out in 2018 and 2019 on 50 milk samples of dairy animals from 50 different localities in Qalyubia and Monufia governorates in the Nile Delta region of Egypt. All dairy cattle and buffalo were local Egyptian breed and kept by smallholders (1–5 animals). They were hand-milked twice daily.

All animals were subjected to clinical examination. Animals with clinical mastitis were defined when one or more of the following signs were observed: cardinal signs of inflammation in one or more of the udder quarters, signs of systemic reaction such as fever, depression and disturbed appetite, and abnormal physical character of milk such as clot formation, discoloration, alterations in viscosity, aberrant smell or presence of blood. Due to the absence of observable clinical signs in animals, the presumptive diagnosis of subclinical mastitis was done based on laboratory diagnostic tests of milk samples, including the California Mastitis Test (CMT).

Milk samples were taken and stored as previously described [11].

Isolation of bacteria from milk samples was carried out as described by the National Mastitis Council [12]. A loopful of milk sample was streaked on blood agar (Oxoid Deutschland GmbH, Wesel, Germany) supplemented with 5% sheep red blood cells and then sub-cultured on selective media: Mannitol Salt Agar, Edwards Medium and Brilliance ESBL Agar (Oxoid Deutschland GmbH) for identification of ESBL-producing microorganisms. All plates were incubated aerobically at 37 °C for 24 h. The plates were examined for colony morphology, pigmentation and hemolytic characteristics after 24 and 48 h.

### 2.2. Matrix-Assisted Laser Desorption/Ionization Time-of-Flight Mass Spectrometry (MALDI-TOF MS)

Isolates were identified using matrix-assisted laser desorption/ionization time-of-flight mass spectrometry (MALDI-TOF MS) [13]. Briefly, bacteria from overnight cultures were suspended in 300 µL of bi-distilled water and mixed with 900 µL of ethanol (96% vol/vol; Carl Roth GmbH, Karlsruhe, Germany) for precipitation. After centrifugation for 5 min at 10,000× *g*, the supernatant was removed, and the pellet was re-suspended in 50 µL of 70% (vol/vol) formic acid (Sigma-Aldrich Chemie GmbH, Steinheim, Germany). Fifty microliters of acetonitrile (Carl Roth GmbH) were added, mixed and centrifuged for 5 min at 10,000× *g*. One and a half microliters of the supernatant were transferred onto a MTP 384 Target Plate Polished Steel TF (Bruker Daltonik GmbH, Bremen, Germany). After air-drying, the material was overlaid with 2 µL of a saturated solution of α-cyano-4-hydroxycinnamic acid (Sigma-Aldrich Chemie GmbH) in a mix of 50% acetonitrile and 2.5% trifluoroacetic acid (Sigma-Aldrich Chemie GmbH). After air-drying, spectra were acquired with an Ultraflex instrument (Bruker Daltonik GmbH). The instrument was calibrated with the IVD Bacterial Test Standard (Bruker Daltonik GmbH). Analysis was carried out with the Biotyper 3.1 software (Bruker Daltonik GmbH). Interpretation of results was performed according to the manufacturer’s recommendation: score of ≥ 2.3 represented reliable species level identification, score 2.0–2.29, probable species level identification, score 1.7–1.9, probable genus level identification, and score ≤ 1.7 was considered an unreliable identification.

### 2.3. Antibiotic Susceptibility Testing using Broth Microdilution

The antibiotic susceptibility testing of all *Enterococcus* isolates was performed with the MICRONAUT system for Gram-positive bacteria (MICRONAUT-S MRSA/GP; Merlin, Bornheim, Germany) according to the manufacturer’s recommendations. It allowed the determination of minimum inhibitory concentrations (MICs) of 22 antimicrobial agents, including ampicillin (β-lactam), cefoxitin (β-lactam; cephamycin), ceftaroline (cephalosporin 5th generation), clindamycin (lincosamide), daptomycin (cyclic lipopeptide), erythromycin (macrolide), erythromycin/clindamycin, fosfomycin (epoxide antibiotic), fusidic acid (steroide antibiotic), gentamicin (aminoglycoside), linezolid (oxazolidinone), moxifloxacin (fluorchinolone 4th generation), mupirocin, oxacillin (β-lactam), penicillin G (β-lactam), rifampicin (ansamycine), synercid (streptogramine), teicoplanin (glycopeptide), tigecycline (glycylcycline), trimethoprim/sulphamethoxazole (trimethoxybenzyl pyrimidine/sulfonamide) and vancomycin (glycopeptide).

The MICRONAUT-S FLI MHK plates allowed the determination of MICs for *E. coli* against 14 antimicrobial agents including AMK (amikacin), AMC (amoxicillin/clavulanic acid), CAZ (ceftazidim), CMP (chloramphenicol), CIP (ciprofloxacin), ERY (erythromycin), GEN (gentamicin), IMP (imipenem), LEV (levofloxacin), PEN (penicillin G), RAM (rifampicin), STR (streptomycin), TET (tetracycline) and T/S (trimethoprim/sulfamethoxazole) in serial dilutions of the antibiotics.

Overnight grown bacteria were suspended in NaCl solution (0.9%) to obtain a turbidity corresponding to a McFarland standard of 0.5 (Dr. Lange, CADAS photometer 30, Berlin, Germany). Three hundred microliters of the suspension were diluted with 11 mL of Mueller–Hinton broth (Oxoid Deutschland GmbH), resulting in a concentration of approximately 10^6^–10^7^ colony forming units (cfu)/mL. In total, 100 µL of the inoculum were given in each well of the plate. After sealing the plates, they were incubated for 18 to 24 h at 37 °C. Reading of plates was done optically. Interpretation was carried out as recommended by the Clinical and Laboratory Standards Institute [14].

### 2.4. Antibiotic Susceptibility Testing using the VITEK 2 System

All *E. coli* isolates suspected as ESBL producers were subsequently confirmed using an automated microdilution system (VITEK 2, bioMérieux Deutschland GmbH, Nürtingen, Germany) according to the instructions of the manufacturer. For this study, the test card AST-N289 (bioMérieux Deutschland GmbH) was used, which included the following antibiotics: piperacillin (PIP), piperacillin/tazobactam (TZP), cefotaxime (CTX), ceftazidime (CAZ), cefepime (FEB), aztreonam (ATM), imipenem (IMP), meropenem (MEM), amikacin (AMK), gentamicin (GEN), tobramycin (TOP), ciprofloxacin (CIP), moxifloxacin (MXF), tigecycline (TGC), fosfomycin (FOS), colistin (CT) and trimethoprim/sulfamethoxazole (T/S).

### 2.5. DNA Extraction and Detection of Resistance-Associated Genes

Genomic DNA was extracted from bacterial cultures using the High Pure PCR Template Purification Kit (Roche Diagnostics, Mannheim, Germany) according to the instructions of the manufacturer.

For *E. coli*, PCR amplifications were carried out for colistin resistance genes (*mcr*-1, *mcr*-2, *mcr*-3, *mcr*-4 and *mcr*-5), erythromycin resistance genes (*erm*(A) and *erm*(B)), macrolide resistance genes (*msr*C), aminoglycoside resistance genes (*aac*6-*aph*2) and tetracycline resistance genes (*tet*K, *tet*L and *tet*M). Primer sequences and target genes are given in Table 1.

Enterococci PCR amplifications (primers, see Table 1) were done for vancomycin resistance genes (*van*A, *van*B and *van*C1), erythromycin resistance genes (*erm*(B), *erm*(A) and *erm*(C)), penicillin resistance gene (*bla*Z), linezolide resistance genes (*optr*A and *cfr*), macrolide resistance gene (*msr*C), aminoglycoside resistance genes (*aac-aphD*), tetracycline resistance genes (*tet*K, *tet*M, *tet*L and *tet*O) and lincosamide resistance genes (*lnu*A and *lnu*D).

PCR products were analyzed by electrophoresis on 2% agarose gel following staining with ethidium bromide and visualizing under UV.

### 2.6. GenoSerotyping, Detection of Antibiotic Resistance and Virulence-Associated Genes of E. coli Isolates by Microarray Analysis

Serotypes of *E. coli* isolates were determined using the *E. coli* SeroGenoTyping AS-1 Kit (Alere Technologies GmbH, Jena, Germany). Five microliters of extracted RNA-free DNA (with a concentration of at least 100 ng/μL) were biotin-labeled by a primer extension amplification using the *E. coli* SeroGenoTyping AS-1 Kit according to the manufacturer’s instructions. The procedures for multiplex labeling, hybridization and data analysis were carried out as described in a previous study [29].

Antimicrobial resistance (AMR) genotypes and resistance-associated genes were ascertained using the CarbDetect AS-2 Kit and *E. coli* PanType AS-2 Kit, respectively (Alere Technologies GmbH). The data were automatically summarized by the “result collector”, a software tool provided by Alere Technologies GmbH. The detection of virulence-associated genes was performed using the *E. coli* PanType AS-2 Kit. Twenty-eight different gene loci connected with resistance to antibiotics and virulence factors associated with adhesion, fimbriae production, secretion systems, SPATE (serine protease auto-transporters), toxins and miscellaneous genes were detected. Analysis was done as described above.

## 3. Results

### 3.1. Bacterial Isolation and Identification by MALDI-TOF MS

In this study, 17 *Enterococcus* isolates (34.0%) were obtained from 50 milk samples of cattle and buffaloes. Identification by MALDI-TOF MS resulted in *Ent. faecalis* (n = 13; 26.0%), *Ent. casseliflavus* (n = 2; 4.0%) and *Ent. hirae* (n = 2; 4.0%). Additionally, 8 coliform isolates (7 *E. coli* and one *Enterobacter cloacae*) could be cultivated. Distribution of isolates from cattle and buffalo is given in Table 2.

### 3.2. Antimicrobial Susceptibility Profiles of Enterococcus Isolates

All *Enterococcus* isolates were examined for their susceptibility to 22 antimicrobial agents. Table 3 shows that all *Ent. faecalis* isolates were resistant to clindamycin, erythromycin, gentamicin, rifampicin, synercid, trimethoprim/sulfamethoxazole and daptomycin. The resistance rate for linezolide, moxifloxacin, erythromycin and others reached 92.3%. Vancomycin resistance rate was high, too (76.9%). Resistance rates regarding other antibiotics ranged between 38.4% for ampicillin and 84.6% for oxacillin.

*Ent. casseliflavus* and *Ent. hirae* isolates were resistant to clindamycin, erythromycin, fosfomycin, fusidic acid, daptomycin, rifampicin, trimethoprim/sulphomethoxazole, oxacillin, moxifloxacin and gentamicin. Resistance rate for vancomycin was 25.0%.

### 3.3. Antimicrobial Susceptibility Profiles of Escherichia coli Isolates

All *E. coli* isolates were resistant to penicillin, streptomycin, erythromycin, chloramphenicol, rifampicin and trimethoprim/sulfamethoxazole (Table 4). Ceftazidim, ciprofloxacin, gentamicin, levofloxacin and tetracycline followed with a resistance rate of 85.7%. Other antibiotics showed resistance rates of 42.9% for amoxicillin/clavulanic acid and imipenem and of 71.4% for amikacin, respectively.

The VITEK 2 system confirmed all 7 *E. coli* isolates as ESBL-producing. They showed resistance to piperacillin, cefotaxime, ceftazidime, astreonam, cefepime, gentamicin and trimethoprim/sulfamethoxazole, as shown in Table 5. All isolates were susceptible to imipenem and meropenem.

### 3.4. Detection of Resistance-Associated Genes in Enterococcus Isolates

All *Ent*. *faecalis* isolates harbored the *erm*(B) gene, associated with erythromycin resistance, the *tet*L gene, connected with tetracycline resistance, and *aac-aph*D, responsible for aminoglycoside resistance (Table 6). Other frequently detected resistance determinants were *bla*Z, associated with penicillin resistance, and *tet*M, associated with tetracycline resistance (84.6%). The *van*A gene was detected in 53.8% of *Ent. faecalis* isolates, while *van*B and *van*C1 genes were not found. Other detected resistance-associated genes were *msrC* (n = 3), *optr*A (n = 2), *lnu*A (n = 2), *lnu*D (n = 1) and *erm*(A) (n = 1).

Other *Enterococcus* (n = 4) isolates exhibited high prevalence of resistance genes *erm*(B), *tet*L, *aac-aph*2 and *van*B, with 100%, 100%, 75.0% and 75.0%, respectively. There were two *Ent. casseliflavus* isolates, one exhibited the *van*A gene and the other one carried the *van*B gene.

### 3.5. Detection of Resistance-Associated Genes in Coliform Bacteria

*E. coli* isolates exhibited high prevalence of the *erm*(B) gene (71.4%), followed by *tet*L (57.1%), while the *tet*K and *msr*C genes, responsible for macrolide resistance, were found only in 2 isolates (28.6%). Aminoglycoside resistance-associated genes *aac*6-*aph*2 were detected only in one isolate (14.3%). No colistin resistance genes have been detected.

In this study, one *Enterobacter cloacae* isolate was examined for the presence of antibiotic resistance genes. It harbored *bla*Z, *erm*(B), *tet*L, *tet*O and *aac*6-*aph*2 genes.

### 3.6. GenoSerotyping and Analysis of Escherichia coli Isolates by Microarray Investigation

Table 7 shows the data detected by microarray analysis. All of the 7 isolates were detected to be *E. coli*. Four isolates were determined by O-serotyping as O8 (n = 1), two were O86 and one isolate was O157. Analysis of three isolates failed. H-serotyping resulted in H11, H12, H21 (two isolates each) and one was of H16 type.

Different virulence-associated genes were detected in *E. coli* isolates, including the fimbrea-associated gene (*lpf*A). Toxin genes *ast*A, *cba*, *cel*B and *cma* were detected in 6 isolates, whereas only isolates 19CS0069 and 19CS0092-1 carried all four determinants (Table 7). Others were carriers of one or two of these genes. Miscellaneous virulence-associated genes like *hem*L and *int*I1 were detected in all isolates, *int*I2 was found in one isolate. Two isolates carried *iro*N and *iss* genes, respectively. No Shiga toxin gene or those responsible for adhesion or secretion systems were detected.

Various antibiotic resistance-associated genes were detected (Table 7). Associated with aminoglycoside resistance, the *aad*A1 gene was identified in 6 isolates, *aad*A4 in 2, *aph*A in 4, *str*A in 5 and *str*B in 3 isolates. The most frequently detected chloramphenicol resistance genes were *cml*A1 and *flo*R, found in 6 isolates. Two isolates carried the *cat*A1 gene. The *mph*A gene responsible for macrolide resistance was detected in 3 isolates. Quinolone resistance-associated genes *qnr*A1 and *qnr*S were found in two isolates. The *tet*A gene connected with tetracycline resistance was found in 3 isolates. Three sulphonamide resistance determinants including *sul*1, *sul*2 and *sul*3 were detected in two, two and 6 isolates respectively, while four different genes associated with trimethoprim resistance were identified, including *dfr*A12 in 3 isolates and *dfr*A17, *dfr*A1 and *dfr*A14 in 2 isolates each.

Genes characteristic for ESBL-producing *E. coli* were detected in all isolates to different degrees: *bla*_CTX-M9_ in 5 isolates, *bla*_CTX-M1, M15_ in 2 isolates and *bla*_TEM_ in 4 isolates.

## 4. Discussion

Raw milk consumption could present a potential risk for public health due to the presence of foodborne pathogens and spoilage bacteria from raw milk samples.

*Enterococcus* species were encountered in studies regarding milk connected with mastitis. In this study, prevalence of *Enterococcus* species in milk of cattle and buffalo was 34.0%, which is similar to a report of the authors of Reference [30], who found that *Enterococcus* species were present in 31.0% of cow milk samples in Iraq.

One of the most important environmental pathogens causing mastitis is *Ent. faecalis*. In this study, the most frequently isolated *Enterococcus* species was *Ent. faecalis*, which is in agreement with other authors’ previous work [30,31,32].

*Ent. faecalis* was found in 26.0% of isolates, similar to other studies that reported the prevalence of *Ent. faecalis* in bovine mastitis cases to be 19.5% in Egypt [33] and 20.9% in Czech Republic [34]. Other enterococci, *Ent. casseliflavus* (4.0%) and *Ent. hirae* (4.0%), were found in similar percentages, as reported in Reference [35].

Enterococci have developed various intrinsic and acquired resistance mechanisms to antibiotics. They have an intrinsic resistance to β-lactams, cephalosporins, clindamycin and low concentrations of aminoglycosides, while acquired resistance to erythromycin, linezolid, daptomycin, tetracyclines, ciprofloxacin and vancomycin was recognized [36].

Vancomycin-resistant enterococci (VRE) were first reported in 1998 and their incidence has increased rapidly through the world after that. Vancomycin-resistance determinants are *van* genes which can be transferred to other Gram-positive bacteria [8]. In this study, phenotypic determined vancomycin resistance was found in 76.9% of *E. faecalis* isolates: 53.8% of them carried the *van*A gene, which is in contrast to results of the authors of Reference [37], who found *E. faecalis* originated from mastitis cases in Turkey resistant to vancomycin at low levels (1.06%).

All *Enterococcus* isolates were found to be phenotypically resistant to erythromycin. This result can be explained by the presence of the *erm*(B) gene in all of them, which is in agreement to reports given by other authors [31,38]. They also found that the *erm*(B) gene was the most prevalent erythromycin resistance gene found in enterococci from both human and animals. Tetracycline resistance in enterococci is often connected with the presence of *tet* genes. Here, *tet*L and *tet*M genes were found frequently, which is nearly in agreement with data reported in Reference [39]. The high percentage of erythromycin and tetracycline resistance found in this and in other studies [38,39,40,41] is due to common and prolonged usage of these antibiotics in the dairy industry for prophylaxis and treatment of mastitis-diseased cattle.

One of the most significant bacterium causing mastitis is *E. coli.* It is isolated in high incidence and it is more dangerous for public health, as a serotype like O157:H7 is enteropathogenic and can cause gastroenteritis, food intoxication, hemorrhagic colitis and hemolytic uremic syndrome. Furthermore, *E. coli* is one of the pathogens that is related to severe clinical mastitis, it is considered as a fatal mastitis pathogen, in some cases it has led to animal death [42]. In this study, in 14.0% of milk samples, *E. coli* was isolated, which is comparable to other Egyptian studies on mastitis-diseased cattle in Egypt with 13.3% and 18.7%, respectively [43,44]. Microarray analysis of *E. coli* isolates revealed that one was serotyped as O157. O157 serotype was reported in some studies in Egypt as a mastitis-causing pathogen [42,43,45] as well as one of the most harmful STEC that can cause severe human infections. Other isolates belonged to serotype O86 within the EPEC group. Isolates of this serogroup were also described as connected with bovine mastitis [46].

ESBL-encoding genes have been categorized into three main types: *bla*_CTX-M_, *bla*_SHV_ and *bla*_TEM_. The *bla*_CTX-M_ isolates have been further categorized into five sub-groups (*bla*_CTX-M-1_, *bla*_CTX-M-2_, *bla*_CTX-M-8_, *bla*_CTX-M-9_, *bla*_CTX-M-25_) and more than 150 variants have been documented (http://www.lahey.org/studies). In our study, all *E. coli* isolates were ESBL-producing, as confirmed by the VITEK 2 system, and this finding was explained by the presence of one or two types of ESBL genes. It agrees with results obtained by several authors for *E. coli* isolates from bovine mastitis cases [5,47,48]. Previous studies suggest that the bla_CTX-M_ type, predominantly bla_CTX-M-15_, was the most prevalent ESBL type worldwide [49]. This result was alarming because *E. coli* which produce ESBLs of bla_CTX-M-15_ type are important causal agents of healthcare-oriented as well as community-based infections in humans [50] and have recently also been increasingly reported from food-producing animals [51]. The cause for frequent occurrence of ESBL-producing bacteria could be the use of β-lactams and even fourth generation cephalosporins in veterinary medicine [52]. Another reason is possibly co-selection by multiple resistance mechanisms through the usage of various antibiotics due to the fact that resistance genes for aminoglycosides, tetracyclines and trimethoprim-sulfamethoxazole are frequently placed on single conjugative plasmids, as is often also the case with *bla*_ESBL_ genes [53]. Generally, ESBL genes are located on plasmids that could spread easily among commensal and pathogenic bacteria within herds and the environment. All ESBL-producing *E. coli* were multidrug-resistant and showed resistance to cephalosporins, β-lactam and non-β-lactam antibiotics, such as ampicillin, aminoglycosides, macrolides, tetracyclines and fluoroquinolones. Many studies confirmed that ESBL-producing *E. coli* were multidrug-resistant independent from the source, like cattle [54,55], poultry [56], pigs [51] and humans [57].

Erythromycin resistance can be connected with the presence of different genes like *erm*(B), *msr*C or *mph*A, which were found in *E. coli* isolates of the milk samples. Similar data have been reported [58]. Other resistance determinants regarding erythromycin resistance played no role.

Phenotypic chloramphenicol resistance was detected in all *E. coli* isolates and DNA microarray analysis resulted in the detection of genes *cml*A1, *flo*R and *cat*A1 responsible for it. Previously, the *flo*R gene was detected in Egyptian *E. coli* isolates from neonatal calves [59] as well as *flo*R and *cml*A in *E. coli* from bovine mastitis cases [60].

Resistance to trimethoprim/sulphamethoxazole was detected in all 7 *E. coli* isolates by both the microdilution method and the VITEK 2 system, which resulted from the presence of sulphonamide resistance genes *sul*1, *sul*2 and *sul*3. The isolates carried at least one or two of these genes, which was already reported previously [61], while carriage of genes *dfr*A1, *dfr*A12, *dfr*A14 and *df*rA17 responsible for resistance to trimethoprim were described for Egyptian isolates from calves and mastitis cases [59,60].

All *E. coli* isolates carried at least one aminoglycoside resistance determinant like *aad*A1, *aad*A4, *aac*6-*aph*2, *aph*A, *str*A or *str*B genes. Similar results have been found previously in *E. coli* isolates from bovine mastitis cases in Egypt [61] and in Iran [62]. Plasmid-mediated quinolone resistance genes *qnr*A1 and *qnr*S were present in only a few isolates, a fact which was reported in the past, too [59,60]. Nevertheless, the detection of these genes is very important due to the possibility of spread of these resistance determinants between bacteria through plasmid mobility [62].

High detection rates of *tet* genes described here were also found in other investigations previously [63,64], in which mainly *tet*A was present in *E. coli* isolates from bovine mastitis cases. Here, the cause of the high presence rate of tetracycline resistance genes is the widespread and uncontrolled usage of this antibiotic for treatment and prevention of infections in livestock in Egypt.

In general, increasing *Enterococcus* and *E. coli* resistance to antibiotics results in excessive use of antimicrobials in different genetic resistance mechanisms, vertically by inheriting genes to new generations or horizontally by exchanging genetic materials among bacteria.

## 5. Conclusions

This study clearly showed that milk from small farmers in Egypt, which is unpasteurized and used as food, is traded or will be processed further, was contaminated with bacteria which are potentially hazardous for human health. To make matters worse, many of these bacteria became multidrug-resistant, whcih makes therapy of resulting diseases hard.

As a consequence, training of farmers is desirable regarding a better hygiene system and pasteurization of produced milk without exception. A surveillance system by governmental institutions should be introduced.

Concerning antibiotic resistance, a reduction of uncontrolled administering of antibiotics should be a primary aim. Only in cases of diseases after serious diagnosis should antimicrobials be used, not as prophylaxis or even as growth promoters. The usage of last-line antibiotics like vancomycin is not allowed in veterinary medicine.

## Figures and Tables

**Table 1:** Primers and their sequences used for the detection of antibiotic resistance-associated genes in *Enterococcus* species and *Escherichia coli* isolates.

Antibiotic	Target Gene	Primer Sequences (5′–3′)	Expected Amplicon Size (bp)	Reference
Methicillin/ oxacillin	*mec*A	F: TCC AGA TTA CAA CTT CAC CAG G R: CCA CTT CAT ATC TTG TAA CG	161	[15]
	*mec*B	F: TTA ACA TAT ACA CCC GCT TG R: TAA AGT TCA TTA GGC ACC TCC	2263	[16]
	*mec*C	AL3: TCA AAT TGA GTT TTT CCA TTA TCA AL4: AAC TTG GTT ATT CAA AGA TGA CGA	1931	[16]
Penicillin	*bla*Z	F: AAG AGA TTT GCC TAT GCT TC R: GCT TGA CCA CTT TTA TCA GC	517	[17]
Vancomycin	*van*A	F: ATG AAT AGA ATA AAA GTT GCA ATA R: CCC CTT TAA CGC TAA TAC GAT CAA	1030	[18]
	*van*B	F: AAG CTA TGC AAG AAG CCA TG R: CCG ACA AAA TCA TCC TC	536	[18]
	*van*C1	F: GGA ATC AAG GAA ACC TC R: CTT CCG CCA TCA TAG CT	822	[19]
Erythromycin	*erm*(B)	F: GAA AAG GTA CTC AAC CAA ATA R: AGT AAC GGT ACT TAA ATT GTT TAC	639	[20]
	*erm*(A)	F: TAT CTT ATC GTT GAG AAG GGA TT R: CTA CAC TTG GCT TAG GAT GAA A	138	[15]
	*erm*(C)	F: CTT CTT GAT CAC GAT AAT TTC C R: ATC TTT TAG CAA ACC CGT ATTC	189	[15]
Macrolide	*msr*C	F: AAG GAA TCC TTC TCT CTC CG R: GTA AAC AAA ATC GTT CCC G	342	[21]
Tetracycline	*tet*K	F: TCG ATA GGA ACA GCA GTA R: CAG CAG ATC CTA CTC CTT	169	[22]
	*tet*L	F: TCG TTA GCG TGC TGT CAT R: GTA TCC CAC CAA TGT AGC CG	267	[22]
	*tet*M	F: GTG GAC AAA GGT ACA ACG AG R: CGG TAA AGT TCG TCA CAC AC	406	[22]
	*tet*O	F: AACTTA GGC ATT CTG GCT CAC R: TCC CAC TGT TCC ATA TCG TCA	515	[22]
Aminoglycoside	*aac*6-*aph*2	F: CCA AGA GCA ATA AGG GCA TA R: CAC TAT CAT AAC CAC TAC CG	219	[23]
	*aac-aphD*	F: TAA TCC AAG AGC AAT AAG GGC R: GCC ACA CTA TCA TAA CCA CTA	227	[15]
Linezolid, chloramphenicol	*optr*A	F: AGG TGG TCA GCG AAC TCA R: ATC AAC TGT TCC CAT TCA	1400	[24]
Oxazolidinone	*cfr*	F: TGA AGT ATA AAG CAG GTT GGG AGT CA R: ACC ATA TAA TTG ACC ACA AGC AGC	400	[24]
Lincosamide	*lnuD*	F: ACG GAG GGA TCA CAT GGT AA R: TCT CTC GCA TAA TAA CCT TAC GTC	475	[25]
	*lnuA*	F: GGT GGC TGG GGG GTA GAT GTA TTA ACT GG R: GCT CTC TTT GAA ATA CAT GGT ATT TTT CGA TC	323	[26]
Colistin	*mcr*-1	F: AGT CCG TTT GTT CTT GTG GC R: AGA TCC TTG GTC TCG GCT TG	320	[27]
	*mcr*-2	F: CAA GTG TGT TGG TCG CAG TT R: TCT AGC CCG ACA AGC ATA CC	715	[27]
	*mcr*-3	F: AAA TAA AAA TTGTTC CGC TTA TG R: AAT GGA GAT CCC CGT TTT T	929	[27]
	*mcr*-4	F: TCA CTT TCA TCA CTG CGT TG R: TTG GTC CAT GAC TAC CAA TG	1116	[27]
	*mcr*-5	F: ATG CGG TTG TCT GCA TTT ATC R: TCA TTG TGG TTG TCC TTT TCT G	1644	[28]

**Table 2:** Prevalence of *Enterocococcus* and coliform isolates in milk samples.

	Origin of Milk	Number of Milk Samples	*Enterococcus faecalis*		*Enterococcus casseliflavus*		*Enterococcus hirae*		*Escherichia coli*		*Enterobacter cloacae*	
			n	%	n	%	n	%	n	%	n	%
Clinical mastitis	Cattle	22	5	22.7	1	4.5	1	4.5	2	9.1	0	0.0
	Buffalo	10	3	30.0	0	0.0	1	10.0	1	10.0	1	10.0
Subclinical mastitis	Cattle	5	3	60.0	1	20.0	0	0.0	2	40.0	0	0.0
	Buffalo	13	2	15.4	0	0.0	0	0.0	2	15.4	0	0.0
Total		50	13	26.0	2	4.0	2	4.0	7	14.0	1	2.0

**Table 3:** Antimicrobial resistance in *Enterococcus* isolates.

Antibiotic	Class	*Enterococcus faecalis* (n = 13)				Other *Enterococcus* Species (n = 4)			
		S	I	R	Resistance Rate (%)	S	I	R	Resistance Rate (%)
Ampicillin	β-Lactam	8	0	5	38.4	2	0	2	50.0
Cefoxitin	β-Lactam; cephamycin	1	0	12	92.3	0	0	4	100
Ceftaroline	Cephalosporin 5th generation	1	0	12	92.3	1	2	1	25.0
Clindamycin	Lincosamide	0	0	13	100	0	0	4	100
Daptomycin	Cyclic lipopeptide	0	0	13	100	0	0	4	100
Erythromycin	Macrolide	0	1	12	92.3	0	0	4	100
Erythromycin/ clindamycin		0	0	13	100	0	0	4	100
Fosfomycin	Epoxide antibiotic	1	0	12	92.3	0	0	4	100
Fusidic acid	Steroide antibiotic	1	0	12	92.3	0	0	4	100
Gentamicin	Aminoglysides	0	0	13	100	0	0	4	100
Gentamicin high level	Aminoglysides	1	0	12	92.3	0	1	3	75.0
Linezolid	Oxazolidinone	1	0	12	92.3	0	1	3	75.0
Moxifloxacin	Fluorchinolone 4th generation	1	0	12	92.3	0	0	4	100
Mupirocin		1	2	10	76.9	0	2	2	50.0
Oxacillin	beta-Lactam	2	0	11	84.6	0	0	4	100
Penicillin G	beta-Lactam	1	5	7	53.8	1	2	1	25.0
Rifampicin	Ansamycine	0	0	13	100	0	0	4	100
Synercid	Streptogramine	0	0	13	100	0	2	2	50.0
Teicoplanin	Glycopeptide	3	0	10	76.9	3	0	1	25
Tigecycline	Glycylcycline	2	0	11	84.6	1	0	3	75.0
Trimethoprim/ sulphamethoxazole	Dihdrofolatreductase/ Sulfonamide	0	0	13	100	0	0	4	100
Vancomycin	Glycopeptide	2	1	10	76.9	2	1	1	25.0

**Table 4:** Phenotypic resistance detected by MICRONAUT system and resistance-associated genes found in of *Escherichia coli* isolates.

Isolate	Phenotypic Antimicrobial Resistance	Detected Resistance-Associated Genes
19CS0095-1	PEN, STR, CAZ, CIP, LEV, GEN, AMK, TET, ERY, CMP, RAM, T/S	*erm*(B), *tet*K
19CS0065	PEN, STR, AMC, CAZ, IMP, ERY, CMP, RAM, T/S	*erm*(B)
19CS0080-1	PEN, STR, AMC, CAZ, IMP, CIP, LEV, GEN, TET, ERY, CMP, RAM, T/S	*tet*L, *tet*K
19CS0092-1	PEN, STR, AMC, CAZ, IMP, CIP, LEV, GEN, AMK, TET, ERY, CMP, RAM, T/S	*erm*(B), *msr*C, *tet*L
19CS0078-1	PEN, STR, CIP, LEV, GEN, AMK, TET, ERY, CMP, RAM, T/S	*erm*(B), *aac*6-*aph*2, *tet*L
19CS0069	PEN, STR, CAZ, CIP, LEV, GEN, AMK, TET, ERY, CMP, AM, T/S	*msr*C
19CS0098-1	PEN, STR, AMP, CAZ, CIP, LEV, GEN, TET, ERY, CMP, RAM, T/S	*erm*(B), *tet*L

AMK (amikacin), AMC (amoxicillin/clavulanic acid), CAZ (ceftazidime), CMP (chloramphe-nicol), CIP (ciprofloxacin), ERY (erythromycin), GEN (gentamicin), IMP (imipenem), LEV (levofloxacin), PEN (penicillin G), RAM (rifampicin), STR (streptomycin), TET (tetracycline), T/S (trimethoprim/sulfamethoxazole).

**Table 5:** Results of antimicrobial resistance test for 7 *E. coli* isolates using the VITEK 2 system.

Isolate	PIP	TZP	CTX	CAZ	FEB	ATM	IMP	MEM	AMK	GEN	TOB	CIP	MXF	TGC	FOS	CT	T/S	
19CS0095-1	R	I	R	R	R	R	S	S	S	R	R	R	R	S	S	S	R	ESBL
19CS0065	R	I	R	R	R	R	S	S	S	S	S	S	S	S	S	S	R	ESBL
19CS0080-1	R	I	R	R	R	R	S	S	S	R	S	S	S	S	S	S	R	ESBL
19CS0092-1	R	I	R	R	R	R	S	S	S	R	R	S	R	S	R	S	R	ESBL
19CS0078-1	R	I	R	R	R	R	S	S	S	R	R	R	R	R	R	R	R	ESBL
19CS0069	R	I	R	R	R	R	S	S	S	R	R	S	R	S	R	S	R	ESBL
19CS0098-1	R	I	R	R	R	R	S	S	S	R	R	R	R	S	S	S	R	ESBL

PIP—piperacillin, TZP—piperacillin/tazobactam, CTX—cefotaxime, CAZ—ceftazidime, FEB—cefepime, ATM—aztreonam, IMP—imipenem, MEM—meropenem, AMK—amikacin, GEN—gentamicin, TOB—tobramycin, CIP—ciprofloxacin, MXF—moxifloxacin, TGC—tigecycline, FOS—fosfomycin, CT—colistin, T/S—trimethoprim/sulfamethoxazole.

**Table 6:** Antibiotic resistance-associated genes detected in *Enterococcus* isolates.

		*Enterococcus faecalis* (n = 13)		Other *Enterococcus* Species (n = 4)	
		Positive (n)	%	Positive (n)	%
Vancomycin resistance genes	*van*A	7	53.8	1	25.0
	*van*B	0	0.0	3	75.0
	*van*C1	0	0.0	1	25.0
Erythromycin resistance genes	*erm*(A)	1	7.7	0	0.0
	*erm*(B)	13	100	4	100
	*erm*(C)	0	0.0	0	0.0
Penicillin resistance gene	*bla*Z	11	84.6	1	25.0
Linezolide resistance genes	*optr*A	2	15.4	0	0.0
	*cfr*	0	0.0	0	0.0
Macrolide resistance gene	*msr*C	3	23.1	0	0.0
Aminoglycoside resistance genes	*aac-aph*D	13	100	3	75.0
Tetracycline resistance genes	*tet*K	0	0.0	0	0.0
	*tet*M	11	84.6	1	25.0
	*tet*L	13	100	4	100
	*tet*O	0	0.0	0	0.0
Lincosamide resistance genes	*lnu*A	2	15.4	0	0.0
	*lnu*D	1	7.7	2	50.0

**Table 7:** Results of DNA microarray analysis of *E. coli* isolates including genoserotyping and detection of genes associated with virulence and antibiotic resistance.

	19CS0065	19CS0069	19CS0078-1	19CS0080-1	19CS0092-1	19CS0095-1	19CS0098-1
*Escherichia coli*	+	+	+	+	+	+	+
*dna*E ^a^	+	+	+	+	+	+	+
*gad* ^a^	+	+	+	+	+	+	+
*gap*A ^a^	-	+	-	+	+	+	+
*ihf*A ^a^	+	+	+	+	+	+	+
*rrs* ^a^	+	+	+	+	+	+	+
O-serotyping	08	086	-	-	086	-	O157
H-serotyping	H11	H12	H21	H11	H12	H16	H21
*lpf*A ^b^	-	-	+	-	-	-	+
*tsh* ^c^	-	+	-	-	+	-	-
astA ^d^	-	+	+	-	+	-	+
*cba* ^d^	-	+	-	-	+	-	-
*cel*B ^d^	-	+	-	-	+	-	-
*cma* ^d^	-	+	+	+	+	+	+
*hem*L ^e^	+	+	+	+	+	+	+
*int*l1/2 ^e^	+	+	+	+	+	+	+
*iro*N ^e^	-	-	-	-	-	+	-
*iss* ^e^	-	-	-	-	-	+	-
blaCTX-M1,M15 ^f^	-	+	-	-	+	-	-
blaTEM ^f^	-	+	+	-	+	+	+
blaCTX-M9 ^f^	+	-	+	+	-	+	+
Aminoglycosides ^g^	*aad*A1, *aad*A4	*aad*A1, *aph*A, *str*A, *str*B	*aad*A1, *aph*A, *strA*	*aad*A1, *aad*A4, *aph*A	*aad*A1, *aph*A, *str*A, *str*B	*str*A, *str*B	*aad*A1, *aph*A, *str*A
Chloramphenicol ^g^	*cml*A1	*cml*A1, *floR*, *cat*A1	*cml*A1, *flo*R	*cml*A1, *flo*R	*cml*A1, *floR*, *cat*A1	*flo*R	*cml*A1, *flo*R
Macrolides ^g^	-	*mph*A	-	-	*mph*A	*mph*A	-
Quinolones ^g^	-	*qnr*A1, *qnr*S	-	-	-	-	*qnr*A1, *qnr*S
Tetracycline ^g^	**-	*tet*A	*tet*A	-	-	*tet*A	-
Sulphonamides ^g^	*sul*3	*sul*1, *sul*2, *sul*3	*sul*1, *sul*3	*sul*3	*sul*3	*sul*2	*sul*3
Trimethoprim ^g^	*dfr*A17	*dfr*A1	*dfr*A12	*dfr*A17	*dfr*A1, *dfr*A14	*dfr*A12	*dfr*A12, *dfr*A14

^a^ Family, genus and species-specific marker, ^b^ Genes encoding virulence factors—fimbriae, ^c^ Genes encoding virulence factors—SPATE, ^d^ Genes encoding virulence factors—toxins, ^e^ Genes encoding virulence factors—miscellaneous, ^f^ ESBL genes, ^g^ Genes associated with antimicrobial resistance.

## Data Availability

Not applicable.
